# Canines (*Canis lupus familiaris*) as biodetectors for conservation work: Can they discriminate the rock ptarmigan (*Lagopus muta*) from the willow grouse (*L*. *lagopus*) in a yes/no task?

**DOI:** 10.1371/journal.pone.0228143

**Published:** 2020-01-28

**Authors:** Charlotte Holmstad Arnesen, Christin Beate Johnsen, Jean-Marc Costanzi, Frank Rosell

**Affiliations:** Faculty of Technology, Natural Science and Environmental Health, University of South-Eastern Norway, Bø i Telemark, Norway; University of Lincoln, UNITED KINGDOM

## Abstract

Alpine and arctic bird populations have shown an unmistakable decrease over the last three decades, and the need for conservation is highly necessary. We investigated the use of five privately-owned dogs (*Canis lupus familiaris*) as a non-invasive tool to determine the presence of rock ptarmigan (*Lagopus muta*), through sniffing out faecal pellets, using a yes/no training regime. We carried out 36 double-blind experimental trials per dog and hypothesised that dogs could discriminate the rock ptarmigan from similar species, such as black grouse (*Tetrao tetrix*), western capercaillie (*T*. *urogallus)* and willow grouse (*L*. *lagopus)*. Our dogs detected differences between the avian species with an average accuracy of 65.9%, sensitivity of 66.7% and specificity of 65.3%. We showed that privately-owned dogs have the potential to be used as biodetectors for conservational work within controlled laboratory conditions for declining species, but overall, only one dog was considered proficient enough. We concluded that dogs could be used as a non-invasive tool to detect the rock ptarmigan, and with further field training and testing, operate in the field for detection surveys.

## Introduction

Alpine and arctic bird populations have shown a global and unmistakable decrease over the last three decades [1, 2]. As there is little knowledge of how alpine bird species will respond to the rapid change in climate, there are reasons to believe several of the effects will potentially be negative [3]. Studies that focus on alpine and arctic bird conservation are therefore highly necessary. The rock ptarmigan (*Lagopus muta*) and the willow grouse (*L*. *lagopus*) are coexisting and sympatric in a large part of their distributional range [4, 5]. The two alpine birds have shown a global declining population trend [6, 7] and the rock ptarmigan is listed as *Near Threatened* in Europe [8]. The species share several morphological traits (e.g. plumage features and colour), as well as cultural traditions for humans as game birds [9]. Usually, the rock ptarmigan occupies habitats in higher altitudes than the willow grouse [4], but in arctic areas, the rock ptarmigan is found breeding at sea level [9]. Potential threats as both climatic changes [10, 11] and disturbance from humans [12, 13] are expected to further increase the possibility for habitat overlap by extending the willow grouse´s habitat to higher altitudes and simultaneously decreasing the rock ptarmigan´s habitat [5]. Currently population monitoring methods have proven to be both time consuming and relatively ineffective [14–16].

Faecal matter does not only provide the researcher a lot of information about the proprietor and its population by observations in the field, such as abundance, habitat use and movement, but also through DNA analysis, such as sex ratio, age, diet, reproductive productivitiy and hormones- and stress levels [17, 18]. Thus, collecting faecal matter is an important and non-invasive method to monitor animal populations. The collection of faecal matter from brown bears (*Ursus arctos)* in Sweden [19] and snow leopards (*Panthera uncia*) in North-Western India, Central China, and Southern Mongolia [20] have helped researchers with monitoring of populations and estimating population size and trends.

A possible non-invasive tool for faecal detection could be the use of dogs (*Canis lupus familiaris*). They have a highly sensitive nose and have been used as field assistants for humans in conservation, research and management for many years [21–23]. Wasser *et al*., [24] compared faecal detection dogs to occupancy-methods by eliciting vocalization responses of the northern spotted (*Strix occidentalis caurina*) and barred (*S*. *varia*) owls. Their result showed that dogs had a significantly higher detection rate than the human-vocalization surveys.

Studies have also shown that dogs can be exceptionally precise in species discrimination with closely related species. Rosell [22] trained dogs in laboratory conditions to discriminate between the two beaver (*Castor spp*.) species, Eurasian beaver (*C*. *fiber*) and the North American beaver (*C*. *canadensis*) via casteoreum collected from dead beavers and scent marks. Smith *et al*., [25] trained detection dogs to recognise faecal matter from San Joaquin kit foxes (*Vulpes macrotis mutica*) and discriminate it from faecal matter from other sympatric fox species and coyotes (*C*. *latrans*).

We investigated the potential to use dogs as a non-invasive conservational tool to determine the presence or absence of the rock ptarmigan through sniffing out faecal pellets in laboratory conditions. We used a yes/no training regime where the dogs were asked to make a yes-decision if faecal pellets from the rock ptarmigan were present, and a no-decision if the faecal pellets from the rock ptarmigan were absent. We hypothesised that the dogs would recognise the rock ptarmigan and discriminate it from the closely related willow grouse, as well as other related species such as black grouse (*Tetrao tetrix*) and western capercaillie (*T*. *urogallus*).

## Material and method

### Scent donors

Species-identified faecal pellets from 85 rock ptarmigans, 85 willow grouse, 20 western capercaillie and 20 black grouse were collected from spring 2015 to winter 2016. Forty-three samples of rock ptarmigan, 43 samples of willow grouse, 20 samples of western capercaillie and 20 samples of black grouse were used in training, and the remaining used in the final experiments (see below). Faecal pellets from the ptarmigans (rock ptarmigan and willow grouse) were collected in 30 different mountain areas in Norway and Sweden in concert with another genetic study and were therefore identified on species- and individual level [26, 27]. Pellet samples from a large number of individual birds from several areas were collected to decrease the chance of dogs recognising and remembering individuals [28, 29] and contribute to a generalised scent impression of the species [30, 31].

Varying quality (different age and degree of degradation through outdoor aging) of faecal pellets was also used, but all pellets were morphologically intact and only used if species-identified. Generalisation of scent samples (different individuals, sex, populations and quality) was important due to the use of aged faecal pellets and to minimise the effect of impure and unknown components within the faecal pellets and to serve dogs with a broader scent picture of the conditioned target [30, 31].

Faecal pellets from the ptarmigans were collected in the field for the genetic study and directly placed in plastic jars (Nalgene, 15 x 38 mm or 30 x 43 mm, Thermo Scientific^™^, Norway). Later, they were taken out of the jars for seven days to dry and cross-contamination of the pellets was avoided by separating them by species and only interacting with them using sterilised equipment [32]. Time from sampling in the field, to when the drying process was initiated, could range from five hours to two weeks. The dry pellets were then inserted into new jars filled with silica gel (Sodium silicate, VWR, BDH Polabo, 28087.361, Norway) and a precision wipe (Kimberly-Clark ^™^, Professional 05511, Norway) to separate the gel from the pellets. The pellets were then stored in a fridge (4°C), and storing time varied from time sampled (spring 2015 to spring 2016), to the time they were added in this study´s sample collection (October 2016). An average of two periods with open lids were carried out per sample (species identification and sex analysis) before use in this study.

For this study, the ptarmigan pellets were weighed (AND Electronic balance FA-200, AC adapter DC 12V 0.3A, China) and placed in glass vials with teflon lids (57 x 27.5 mm, Qorpak®, Pennsylvania, USA) with a mean weight of 0.7 grams (SD ± 0.078). Each sample was handled with a new pair of disposable gloves and sterilised tweezers. They were stored in a freezer (– 20°C) until they were used in either training or experiments.

Collection of faecal pellets from black grouse and western capercaillie was independent from the ptarmigans and was carried out between October and December 2016. They were collected throughout four lowland, forest areas in Telemark and Buskerud county, Norway. None of the ptarmigans had been reported as present in those areas. The pellets were dried for seven days before being inserted into glass vials, and then immediately stored in the freezer (-200°C).

### Dogs and laboratory

Five privately-owned dogs with a mean age of 7.4 years (SD ± 4.07) and a basic level of obedience were used in training (Table 1). Two non-professional female dog handlers with a scientific background handled the dogs. One handler (B) and four dogs had been used in earlier scent detection work [22] (Table 1).

**Table 1:** Subject information. The dogs used, their sex (F = female, M = male), age (year) at first training session, breed, handler, ownership with their handler, scent detection experience and total number of training sessions.

Dog	Sex	Age	Breed	Handler	Ownership	Scent detection experience	Tot number of training sessions
**Akira**	F	1.5	Grosspitz	A	Yes	No	76
**Bailey**	M	5	Nova Scotia duck tolling retriever	B	Yes	Yes	79
**Chilli**	F	9	Border collie	A	No	Yes	83
**Shib**	F	11.5	Border collie	B	No	Yes	83
**Tapas**	M	9	Border collie	A	No	Yes	27

Training took place at the dog laboratory (3.40m x 5.65m, height = 2.1m) at the University of South-Eastern Norway, Bø in Telemark county (59.40978°N and 9.0567698°E), and was carried out from the 25^th^ of October 2016 to 5^th^ of January 2018. Each dog was trained periodically with between 0–3 sessions a week. The duration of a training session was approximately 15–20 minutes per dog.

### Training procedures

Training was split into three phases: 1) adaption to the laboratory and scent imprinting on the table platform, 2) discrimination training, and 3) adaption to a yes/no training regime. All training was exclusively based on positive reinforcement through operant conditioning by use of a clicker-sound as the secondary reinforcer and receival of each dog’s preferred reward (food treats, praise and/or toy, based on previous scent work [22] and motivation)[33–35]. All dogs were familiar with the sound of a click and receiving of a reward.

A table platform adapted in Hällefors, Sweden, by the Hundcampus training center was used in all training phases. Some of the perks of using this platform is to present dogs with target and control scents at the same time (scent discrimination) with little influence from the handler. The table platform is an apparatus that combines a stainless steel formation which is easy to keep clean and plexi glass which serves as a presenter of scents. The platform consists of a movable sledge which holds seven scent locations, one target location, and six control scent locations. A handle is connected to the target scent location on the sledge, which enables the handler to always know of its whereabouts. The handle is placed behind the platform, where the handler is also standing. A plexi glass is connected to the sledge and presents the dog with four scent locations by physically fencing the four scent locations. The sledge can be moved back and forth due to handle, and the sample presenter is always stable, thus the four presented scents vary due the location of the target scent. The sledge is also connected to another stainless steel formation that function as a sample cover so no visual cues can be given to both dog and handler, also including a small wall that covers the arms of the handler so the dog cannot detect target location due to the arm position of the handler (see [36] and [37] for more details).

To emphasis that the dogs learnt to both recognise and discriminate the rock ptarmigan from other odours, a collection of control samples were used in the platform [29, 32, 38]. Faecal pellets from birds in the same family (*Tetraonidae*) and the same genus (*Lagopus*) were used, and other laboratory environmental control samples used during the preparation of samples (e.g. disposable gloves and silica gel).

Since the table platform presents the dogs with four scent options in each lineup, one target scent and three control scents were always present. Therefore, a dog can respond to a sample in four different ways: 1) a true positive (TP) response, the dog lies down in front of the target scent, 2) a false positive (FP) response, the dog lies down in front of a control scent, 3) a true negative (TN) response, the dog correctly rejects control scents and 4) a false negative (FN) response, the dog falsely rejects a target scent [39, 40].

To avoid (cross) contamination, all samples were placed in sterilised plastic cups and disposable gloves were worn at all times when interacting with the samples [41, 42]. The platform was cleaned with a 1:3 water-7% vinegar solution between each dog [43–45], minimising residual odours, saliva and other possible disturbances or cues from other dogs or handlers [46].

#### Ethics statement

All methods were performed in accordance with the relevant guidelines and regulations of the University of South-Eastern Norway and no further permits for pet animals were required. Approvals from other ethics committees or ethics boards were not needed. No animals experienced anaesthesia, euthanasia or any kind of sacrifice as a part of this study. All dogs that contributed to this study had permission obtained from the owner.

No authorisations were needed to 1) access any of the areas for sample collection or 2) collect the faecal pellets. The fecal pellets were non-invasively collected so no animals were disturbed. Thus, no permits or authorisations were needed.

#### Phase one

Phase one was split into two main goals: 1) adaption to the laboratory and table platform, and 2) scent imprinting. In this phase (and phase two), a training session consisted of five trials, and each trial of ten randomly chosen lineups per dog using a random number generator (Microsoft® Excel, Version: 16.16.3, 2016). Thus, one session equals five trials and therefore fifty lineups. The dogs were always presented with one target sample among three control samples in every trial.

Only one dog (Akira) underwent goal one, as she was naïve to the environment, any kind of scent detection work and the table platform. The remaining four dogs had previously been taught the passive response (lay down in front of a target scent) as the trained final response (TFR) [34, 47] and started directly on goal two. To teach Akira to perform a TFR, a dog treat was placed inside the platform and the handler simply started with the command “search” and pointed in the direction of the samples. When the dog started to sniff each hole and discovered the treat scent (target scent), there was an instant change in behaviour. In the beginning, it was tolerated that the dog was pointing with its nose and standing still in the direction of the sample, but after a few correct responses, the handler told the dog “down”, resulting in a click and a following reward. Inserting laboratory control scents such as silica gel, disposable gloves and empty samples glasses in the remaining scent locations the dog associated the treat scent with laying down, as no behaviour on control scents were rewarded. The TFR was later expected to last for at least three seconds [36]. After the dog independently sniffed all samples in the platform and performed 100% correct in three consecutive sessions, the adaption was completed, and the dog carried on to the next goal.

The same method was used for the scent imprinting in goal number two, but here the dog treat was replaced with faecal pellets from rock ptarmigan. The handler simply started with the command “Search” and pointed in the direction of the samples. When the dog was sniffing the target scent, the handler clicked with the clicker and rewarded the dog. This procedure was repeated a few times with varying locations of the target scent until eventually, the dog was laying down in front of it. The scent imprinting was completed when all dogs performed with at least ≥ 80% correct responses (accuracy [48]) in three consecutive sessions [36].

#### Phase two

After the dogs successfully discriminated rock ptarmigan from the laboratory controls scents, phase two continued with the discrimination training. For goal one, the level of difficulty was increased by adding faecal pellets from western capercaillie and black grouse. When the dogs performed with at least ≥ 80% correct responses in five consecutive sessions, faecal pellets from willow grouse were added (goal two). The number of sessions with a least > 80% correct responses were expanded from three to five (three in phase one) to further decrease the possibility of correctness by chance. Phase two was completed when the dogs recognised and discriminated rock ptarmigan from western capercaillie, black grouse and willow grouse with at least ≥ 80% correct responses in five consecutive sessions. One dog (Tapas) did not continue training due to sickness.

#### Phase three

Phase three was an incremental adaption from the table platform to a one-holed platform. The phase was split into four goals: 1) teaching of a TN response, 2) training on a four-holed platform, 3) training on a two-holed platform and 4) training on a one-holed platform. From here on, one training session now consisted of six trials, but the number of scents in the lineup in each trial varied in relation to the goal. Thus, in goal two, the dogs searched four samples in each trial; in goal three the dogs searched two samples in each trial, and in goal four, the dogs searched one sample in each trial.

In addition to the already taught yes-decision (lie down), the dogs were trained to make a no-decision, return to the handler when target was absent [49, 50]. Thus, the dogs FP, TN and FN responds were different from phase one and two: FP response, the dogs lie down in front of a control scent or when target is absent in a trial, TN response, the dog returns to handler when target is absent, and FN response, the dog returns to handler when target is present [39, 40].

Trials when target scent was absent, was implemented in training so the dogs would experience higher credibility since they were trained to not always expect target presence in a search, thus returning to the handler in the absence of the target scent [51]. Such zero trials could consist of a lineup with only blanks (nothing) or with faecal pellets from the willow grouse. To teach the dogs a TN response, an empty table platform (i.e. no scent samples present) was used. The handler was placed behind the dogs, sending them to the platform without the possibility to visually cue them [52]. The handler sent the dog to sniff the platform, and when no response was made the handler called the dog, resulting in a click and a reward when the dog returned. To increase the dogs’ motivation and for them to feel more successful, the trained no-decision was equally rewarded as the yes-decision [49, 53]. At the beginning of training a zero trial consisted only of blank, and later blanks and willow grouse faecal pellets, or only willow grouse faecal pellets. After 10–12 training sessions adapting to the training regime, all dogs learnt the context of lying down in front of the platform when faecal pellets of the rock ptarmigan was present and returning to handler when absent.

Goal two continued after the dogs had learnt the no-decision. Target presence in a trial randomly varied throughout this phase, but there was never more than one target scent present in each trial. From here on, the dog-handlers were blind to the presence and position of the target scent as an experimenter placed the samples. The experimenter was situated in the room next door watching the dog-handler team through a video monitor connected to cameras in the laboratory, hence no potential cuing to the dog-handler team and double-blind training [32, 54]. The experimenter evaluated the team´s responses, and if a correct response was made, a click from a clicker held by the experimenter that could be heard across the rooms was provided, and the dog rewarded. If the team was incorrect, no click was provided and the team exited the laboratory. By using this kind of reward-scenario, we were able to directly reinforce the dog when it performed a correct response, and no reward when incorrect [29, 55].

The same procedures as above were followed in goal three and four, but the table platform was replaced with a two-holed platform and a one-holed platform, respectively. In goal three, the dogs had to chose between two scent alternatives, and in goal four, the dogs had to make a yes- or a no-decision in each trial to determine the presence or absence of the rock ptarmigan [38, 49]. Target presence still varied randomly throughout the training phases, and all training was carried out in a double-blind manner [54]. Because time was limited during phase three, we required ≥ 70% correct responses in five sessions to move on to the final experiments.

### Final experiments

Final experiments were undertaken within an eight weeks period between the 10^th^ of January to 14^th^ of March 2018, and six experimental sessions (36 trials per dog) were carried out. Experimental procedures were equal to the last goal in phase three. All scent samples were naïve to the dogs and only used once. Equal numbers of positive and negative samples were used to ensure that the possibility of a sample to be a target sample was 50%, but varying numbers of positive and negative samples in a session were used, so the dog-handler could not know the target presence.

As a reassurance for any bias caused by potential cuing from the experimenter, a third person, an observer not involved in the study, chose both scent samples and the order of them using a random number generator (Microsoft® Excel, Version: 16.16.3, 2016) [54, 56]. The dog handler team was placed in the training room and directly watched by the experimenter in the second room. The experimenter had telephone contact with the observer, (situated outside of the laboratory) so the experimenter could provide a confirmational click to the handler so the dog could be directly rewarded if correct response was made. All trials were recorded (Sony Handycam DCR-SR, USA) and the tapes were watched and responses confirmed by the observer after the experiments were done to ensure no observational bias [54, 56].

### Data analysis

Three parameters were calculated to evaluate all dogs from the four possible responses: sensitivity, specificity and accuracy [48]:

calculation of sensitivity: TP / (TP + FN),

calculation of specificity: TN / (TN + FP) and

calculation of accuracy: (TP + TN) / (TP + FP + TN + FN).

A Fishers exact test was run to establish if the dogs´ responses (pooled and individual response) were significantly (p < 0.05) better than expected by chance (R, Version 3.1.1).

## Result

### Training

All dogs but one (Tapas) completed all goals and phases within the thresholds values with an average accuracy of 94.6% (SD ± 7.6) in phase one, 91.9% (SD ± 1.8) in phase two, and 75.8% (SD ± 5.6) in phase three (Table 2).

**Table 2:** Training results. Experimental dogs, training phases (one, two, three) and goals, average number of training sessions in each phase, result presented as accuracy in percent from all dogs in all training phases and goals, and pooled accuracy for each phase. Percentages were calculated from the five last consecutive sessions in each goal and phase (except phase one, goal one, where the result was calculated from the result achieved in the last three consecutive sessions).

	Akira	Bailey	Chilli	Shib	Tapas	Average accuracy	Average number of training sessions
**Phase one**	**Adaption to the laboratory and scent imprinting on the table platform**						
Goal one	Adaption to the laboratory and table platform						
Accuracy	100	-	-	-	-	100	6
Goal two	Scent imprinting (rock ptarmigan)						
Accuracy	96.9	94.3	88.0	82.8	84.3	89.3	3.8
***Pooled accuracy***	**94.6**						
**Phase two**	**Discrimination training**						
Goal one	Discriminating target scent (rock ptarmigan) from control scents (western capercaillie and black grouse)						
Accuracy	95.2	93.4	95.6	90.8	91.0	93.2	8.2
Goal two	Discriminating target scent (rock ptarmigan) from control scents (western capercaillie, black grouse and willow grouse)						
Accuracy	90.0	89.8	93.2	91.4	90.4	90.9	15.4
***Pooled accuracy***	**91.9**						
**Phase three**	**Adaption to a yes/no training regime**						
All goals	Teaching of a TN-response, training on a four-holed platform, training on a two-holed platform and training on a one-holed platform						
Accuracy	71.3	73.1	84.0	74.8	-	75.8	50.8
***Pooled accuracy***	**75.8**						

### Final experiments

Each dog carried out six double-blind and randomised sessions (36 trials). They recognised and discriminated the rock ptarmigans from the controls scents with an average accuracy of 65.9% (SD ± 9.7), sensitivity of 66.7% (SD ± 26.1) and specificity of 65.3% (SD ± 18.4). The result revealed a considerable range between the poorest and strongest performing dog in sensitivity (33% and 94%), specificity (44% and 89%) and accuracy (61% and 81%) respectively (Table 3).

**Table 3:** Experiment results. Result are presented as sensitivity, specificity and accuracy in percent for each dog, their pooled average, number of responses from the four evaluated dogs calculated from 36 trials divided in six experimental sessions and P-values calculated from a Fishers exact test. TP = true positive response, FP = false positive response, TN = true negative response, FN = false negative response, CR = correct responses, IR = incorrect responses.

Dog	Trials	TP	FP	TN	FN	CR	IR	Sensitivity	Specificity	Accuracy	P
**Akira**	36	14	10	8	4	22	14	77.8	44.4	61.1	0.8272
**Bailey**	36	6	2	16	12	22	14	33.3	89.9	61.1	0.2702
**Chilli**	36	13	6	9	8	22	14	61.1	61.1	61.1	0.5111
**Shib**	36	17	6	12	1	29	7	94.4	66.7	80.6	0.023
**Total**	**144**	**50**	**24**	**45**	**25**	**95**	**49**	**-**	**-**	**-**	**-**
**Pooled average**								**66.7**	**65.3**	**65.9**	**-**

Overall, our dogs performed significantly better than expected by chance (p = 0.0085). Looking at the individual-basis, there was only one dog (Shib), that performed significantly better than expected by chance (Table 3).

## Discussion

Our result shows that our dogs were able to recognise faecal pellets from the rock ptarmigan and discriminate it from the willow grouse and other related birds significantly better than expected by chance. Although, only one dog was significantly capable of doing so looking at the individual-basis.

This study reveals that there must be a difference in the chemical composition of faecal pellets from the closely related avian species. Both species inhabit slightly similar habitats and may experience habitat overlap [5, 9]. They consume almost similar food, with an exception of the winter diets if both the rock ptarmigan and the willow grouse occur sympatrically [9]. The most suitable explanation would therefore be the difference in their genetics [57, 58].

Several factors can influence a dog-handler team´s accuracy rate [32, 49, 53]. Working dogs have been stated to have increased success when it comes to careful selection in relation to breed and personality traits as e.g. high trainability, high drive (hunt-, prey-, play- and motivational drive), a useful amount of independence, and fitness [34, 49, 59, 60]. An important factor to document, as many of today’s studies are based on highly successful working dogs with long experience, and a random dog may not be capable of achieving their detection skills [25, 30, 39, 49]. None of our dogs were selected in case of personality traits, but rather as convenience. We experienced especially high work ethic and motivational drive with one dog (Shib), which can be a possible explanation to why this dog performed better than the other three dogs. A study has also shown that pet dogs demonstrated higher dependencies towards their owners when it came to novel situations or problem solving compared to exclusive working dogs (dogs who did not live with their caregivers) [61]. However, studies using privately owned pet dogs have also shown successful detection rates with high accuracy [28, 36, 50].

This study reveals a higher false positive and false negative rate than ideal. Working with time constraints, inexperienced handlers and dogs not selected for their desirable working traits may cause several issues [25, 47]. TP- and TN-responses were rewarded as described in Gadbois & Reeve [49], Fischer-Tenhagen *et al*., [50] and Johnen *et al*., [53]. Using a yes/no training regime, there is a 50% chance of a randomly selected sample to be correct and just by guessing, the dogs would have a considerable chance of getting a reward. Fischer-Tenhagen *et al*., [36] stated that dogs that are used with clicker-based training, may in novel situations, perform different kinds of behaviours to get a reward. Hurt *et al*., [47] addressed issues as “lying”, often seen with dogs lacking ideal work ethic or training endurance, which result in a higher false alert rate, where the dogs are skipping the hard part of the training to get to the rewards. Target confusion may also be a possible issue for high false positive and false negative rates generally caused by an insufficient amount of training, where the dogs do not know how to generalise all variants of the target [47]. The sample collection in this study consisted of faecal pellets that had been dried and stored in a fridge with silica gel for up to one and a half years before they were used in dog training, and there was no knowledge on how long the pellets had been outside before they were collected. Including fresh scent samples to the sample collection would have been ideal. However, the importance of generalisation of the target scent was strongly emphasised because of those factors. Handler errors, such as cuing or misreading the dog´s behaviour may also cause the detection dogs and team to fail the search and is usually seen with inexperienced handlers [34]. Our results showed a decrease in the result between phase three (75,8%) and the final experiment (65,9%). Since the training/test regime was identical, except that an observer chose and randomly placed the samples in the platform wearing disposable gloves, a plausible explanation may be that the dogs sensed the non-professional dog handlers state of mind like e.g. nervousness or unintentional dissapointedness when the dogs performed incorrect. Numerous studies have shown that dogs are especially well adapted to humans, their expressions and emotions and they are usually very eager to please their handler/owner [53, 61–65].

Results in training phase three and the final experiment indicates that training on a one-alternative choice platform with a yes/no-decision seemed more challenging for our dogs than training on a multi-alternative choice platform (training phase one and two), also stated in Gadbois and Reeve [49]. A one-alternative choice set up will increase the difficulty of the detection task simultaneously as it will give a good overview of the bias and how to minimize it [49]. A multi-alternative choice set up with more than three alternatives has been stated to increase the sensory and mnemonic interference in dogs and by that decrease their performance [38, 49, 50]. Also, there are speculations of dogs using control scents in the lineup to compare samples to find the target [50]. When using a scent platform, there will always be a possibility that the dogs are learning the specific target sample already after a few encounters, causing dogs not to learn the generalised odour perception of the target, but rather individual odours present in the lineup [36]. In a yes/no training regime, dogs will be presented only one sample at the time with no other scents to compare it with, making it a pure detection task [49, 50]. Comparing our results on the one-alternative choice platform with other previous studies, the average detection accuracy, sensitivity and specificity rate of 73.8%, 56.5% and 91.5% [49] and 75%, 72% and 84% respectively [50] are similar to ours. However, if you are aware of each regime´s (one-alternative choice and multi-alternative choice) benefits and disadvantages, the two scent detection approaches can complement each other and prepare dogs to work in both field and laboratory conditions. While the multi-alternative choice training regime serves the dogs with discrimination tasks they are likely to experience in field (detect faecal pellets from rock ptarmigan and discriminate it from other species), the one-alternative training regime serves the dogs with a pure detection task, and the researcher with dogs that simply decides if the rock ptarmigan is present, so the sample can be sent for further DNA analysis.

To the best of our knowledge, no study has investigated the potential use of dogs to discriminate between avian species in the same genus, and few studies have investigated the potential of dogs to discriminate between mammals within the same genus. Smith *et al*., [25] trained dogs to detect faecal matter from the endangered San Joaquin kit fox and discriminate it from other sympatric fox species and coyotes. Their dogs successfully discriminated the kit fox from the other fox species with an accuracy of 100%, but was less accurate (67%) to ignore the red fox (*Vulpes vulpes*) when the kit fox was absent. Their results cannot be directly compared to ours due to the use of scent detection task, as they used a multi-alternative choice set up, but they state that all the tested dogs performed significantly better when both species were present in the scent lineup. Rosell [22] trained dogs in laboratory conditions to discriminate between the two beaver species, the Eurasian beaver and the North American beaver. They trained dogs using 15-year-old castoreum samples collected from dead beavers (sensitivity = 90% and specificity = 98%). As a second olfactory test, they investigated the potential of dogs to recognise and discriminate the beavers’ scent marks collected in field with high detection accuracy (Sensitivity = 85% and specificity = 94%). They also successfully demonstrated that samples can be brought from the field and into the laboratory for further research. However, they did not use a yes/no training regime, so the results cannot directly be compared to our results.

The collection of faecal pellets can provide the researcher with a lot of information about the species/population investigated [17, 18, 30]. Ways of collecting these samples have proven to be time-consuming, costly and relatively inefficient, simultaneously requiring an additional amount of time in the laboratory after collecting the samples to complete the analysis. Studies have shown dog-human teams to decrease bias, be more accurate and efficient to find targets in the field in relation to time and area (size) searched compared to human-only teams [30, 66, 67]. Dogs have also proven to be more successful in detection compared to hair snares and cameras [68]. Even though our dogs are trained in laboratory conditions, with some further field training including e.g. distractability training and handler control, there is no reason to believe that they cannot be surveying in the field as a non-invasive tool to find faecal pellets from the rock ptarmigan. It is documented that dogs were able to find target in field after laboratory training [38]. However, some dogs have shown difficulties being brought back to the laboratory after high stimulated field search, because of lack of motivation [38] or frustration [25].

As the climate is gradually changing [10] and human disturbances increases [13, 69, 70], the rock ptarmigan and the willow grouse are expected to share a greater amount of habitat [5, 9, 12, 13]. The most accurate method to distinguish the rock ptarmigan from the willow grouse by faecal pellets is identification of species through DNA-analysis, a method considered to be both time-consuming and expensive. Smith *et al*., [25] showed that dogs can reduce both time and cost in species-identification as the result is immediate. In their case, they had the potential to generate savings of approximately $6000 by letting the dogs identify the faecal matter before further DNA-analysis. Bringing field samples into the laboratory is a well known phenomenon in mine detection dogs and other situations where dogs and handler may be in danger entering the field [38, 39]. The method has proven to give many advantages, such as controlled microclimate, optimised scent perception by creating convenient reward strategies and controlled and familiar environment [38]. However, training detection dogs is also time-consuming and expensive, but when they are proficiently trained, little maintenance is required, once a week has proven to be enough [71]. Partnership with public agencies, universities for educational purposes or with e.g. police, could reduce the cost of a trained detection dog dramatically, as the dog already is trained with scent in focus [72].

We showed that dogs have the potential to be used as biodetectors for conservation work within controlled laboratory conditions for the declining species. Our privately-owned dogs were able to discriminate rock ptarmigan from other related species. However, only one dog was considered to be a pre-scanner of faecal pellets to identify the rock ptarmigan before further DNA analysis in the laboratory using a yes/no training regime. We therefore consider personality traits to be of importance when selecting dogs for detection work, as only one out of four dogs were considered to be proficient, even though all dogs followed a systematic training approach. Since our result is based on training in a controlled environment, the next step should be to conduct training outside in uncontrolled environments with varying weather conditions and habitats including distractabiltiy training. Further laboratory training should also be conducted for the remaining dogs. We conclude that dogs may be an efficient non-invasive conservation tool to help manage threatened and vulnerable species.
